# A Multi-Site Prospective Study of Paradoxical Lucidity in Moderate to Severe Dementia

**DOI:** 10.1093/geroni/igaf122.2914

**Published:** 2025-12-31

**Authors:** Maria Tollock, Natalia Leontovich, Anelly Gonzalez, Sam Parnia

**Affiliations:** New York University Langone Health, New York, New York, United States; New York University Langone Health, New York, New York, United States; New York University Langone Health, New York, New York, United States; New York University Langone Health, New York, New York, United States

## Abstract

**Background:**

Paradoxical episodes of lucidity (paradoxical lucidity [PL]/terminal lucidity) especially near the end of life have been reported in patients with severe dementia. Although little is known about PL, its occurrence challenges current assumptions about dementia and highlights the possibility of a network-level return of cognitive function despite severe dementia.

**Methods:**

We conducted the first prospective investigation of PL among severe dementia patients. Among 1,768 patients at NYU Langone, VNS Health, and Bellevue Hospital, 1,405 (79.5%) met inclusion criteria [moderate/severe dementia, life expectancy < 12months]. Of these, 151 (10.7%) were enrolled, 823 (58.6%) declined, 239 (17.0%) were already deceased, and 192 (13.7%) didn’t respond to attempted contacts. Primary caregivers completed a log to report instances of perceived mental clarity.

**Results:**

Of 151, 93 (61.6%) reported lucidity, with 267 distinct events that were consistent with reality: 1) appropriate orientation to events (67.8%), 2) return of old memories (34.8%), 3) return of functional abilities (27.7%), 4) nonverbal communication (25.1%), 5) terminal lucidity (memories consistent with near-death experiences) (4.1%). A further 9.7% reported hallucinations/delusions, which were not consistent with reality. Potential triggers to PL episodes were identified: music, anniversaries, emotional distress, and medication changes, including immunomodulating agents and antihistamines.

**Conclusion:**

Multiple types of sudden paradoxical lucid events occur in end-stage dementia. These can be triggered by multiple factors including medications. PL is common and may provide opportunities for future therapeutic interventions. Research is needed to identify the triggers and underlying mechanisms driving PL.

